# Electrically evoked compound action potentials are associated with the site of intracochlear stimulation

**DOI:** 10.1007/s00405-024-08493-4

**Published:** 2024-03-29

**Authors:** Nora M. Weiss, Tabita Breitsprecher, Christiane Völter, Marc Lammers, Paul Van de Heyning, Stefan Dazert, Vincent Van Rompaey

**Affiliations:** 1 Department of Otorhinolaryngology-Head and Neck Surgery, Ruhr-University Bochum, St. Elisabeth-Hospital Bochum, Bochum, Germany; 2 Department of Translational Neurosciences, Faculty of Medicine and Health Sciences, University of Antwerp, Antwerp, Belgium; 3 International Graduate School of Neuroscience (IGSN), Ruhr-University Bochum, Bochum, Germany; 4 Department of Otorhinolaryngology and Head and Neck Surgery, Antwerp University Hospital, Antwerp, Belgium

**Keywords:** ECAP characteristics, Electrode position, Anatomy-based fitting, Patient-individualized cochlear implantation

## Abstract

**Objectives:**

Objective measurements to predict the position of a cochlear electrode during cochlear implantation surgery may serve to improve the surgical technique and postoperative speech outcome. There is evidence that electrically evoked compound action potentials (ECAP) are a suitable approach to provide information about the site of stimulation. This study aims to contribute to the knowledge about the association between the intraoperative intracochlear ECAP characteristics and the site of stimulation.

**Methods:**

In a retrospective cohort study, patients undergoing cochlear implant surgery with flexible lateral wall electrode arrays (12 stimulating channels) between 2020 and 2022 were analyzed. The CDL was measured using a CT-based clinical planning software. ECAP were measured for all electrode contacts and associated to the CDL as well as to the site of stimulation in degree.

**Results:**

Significant differences among the amplitudes and slopes for the individual stimulated electrode contacts at the stimulation sites of 90°, 180°, 270°, 360°, 450° and 540° were found. The values showed a trend for linearity among the single electrodes.

**Conclusions:**

ECAP characteristics correlate with the electrode’s position inside the cochlea. In the future, ECAP may be applied to assess the intracochlear position inside the cochlea and support anatomy-based fitting.

## Introduction

Recently, objective measurement methods are increasingly becoming topics of interest in the treatment of patients with severe to profound hearing loss with a cochlear implant (CI), especially with regard to implant fitting in children or patients with poor compliance [1–4]. Furthermore, in this context, the position of the electrode and the angle of insertion are of interest for improving the hearing perception outcome [5]. The electrically evoked compound action potentials (ECAP) are considered a promising approach for CI fitting [6, 7] especially in children, since they are able to provide objective information about the stimulation thresholds. There is evidence that ECAP are also suitable to provide information not only about the neural integrity of spiral ganglion neurons but also about the site of stimulation [8–10]. The electrode position inside the cochlear is of interest for anatomy-based fitting approaches that have been shown to provide improved speech perception [11, 12]. However, the size of the human cochlea can significantly affect the CI electrode position within the cochlea and consequently structure preservation as well as the final pitch discrimination [13, 14]. Consequently, choosing the electrode variant fitting the individual expectations is highly relevant. To predict the postoperative electrode position from preoperative clinical imaging, it has been demonstrated that a clinical planning software for measuring the human cochlea (Otoplan, Cascination, Bern, Switzerland) is adequate to determine the length of the cochlear duct and to select the electrode length according to the aimed insertion depth [15]. However, it has been shown that there are inaccuracies concerning the insertion depth prediction of the software [16]. Thus, a feedback mechanism providing intraoperative information about the insertion depth is desirable. Thus, the aim of this work was to determine reference values for ECAP characteristics at different locations inside the cochlea. This study aims to contribute to the knowledge about the association between changing ECAP characteristics inside the cochlea according to the CDL.

## Methods

### Patients selection

Between 2020 and 2022, patients scheduled for cochlear implant surgery due to severe and profound hearing loss were assessed for inclusion. Patients implanted with flexible lateral wall electrode arrays with 12 stimulating channels (Med-el GmbH, Innsbruck, Austria) without functional residual hearing were included into the study. Patients with insufficient imaging quality to identify the postoperative electrode position were excluded. The study protocol was approved by the local Ethics Committees in accordance with the Helsinki declaration.

### Preoperative determination of cochlear duct length and estimation of cochlear coverage

All measurements were performed using an otosurgical planning software (Otoplan, Cascination AG, Bern, Switzerland). The pre-defined anatomical landmarks (the round window and lateral wall of the cochlea) to determine the diameter (*A* value) and the width (*B* value: perpendicular to the line segment of the *A* value, intersecting the *A* value line at the modiolus) of the cochlea were marked by a single investigator experienced in the interpretation of temporal bone imaging. The calculation of the CDL approximation is performed by the software based on an elliptic circular approximation [29]. The electrode length was chosen to cover 75% of the CDL according to the preoperative CDL estimation.

### Postperative determination of insertion angle and cochlear coverage

**Fig. 1:**
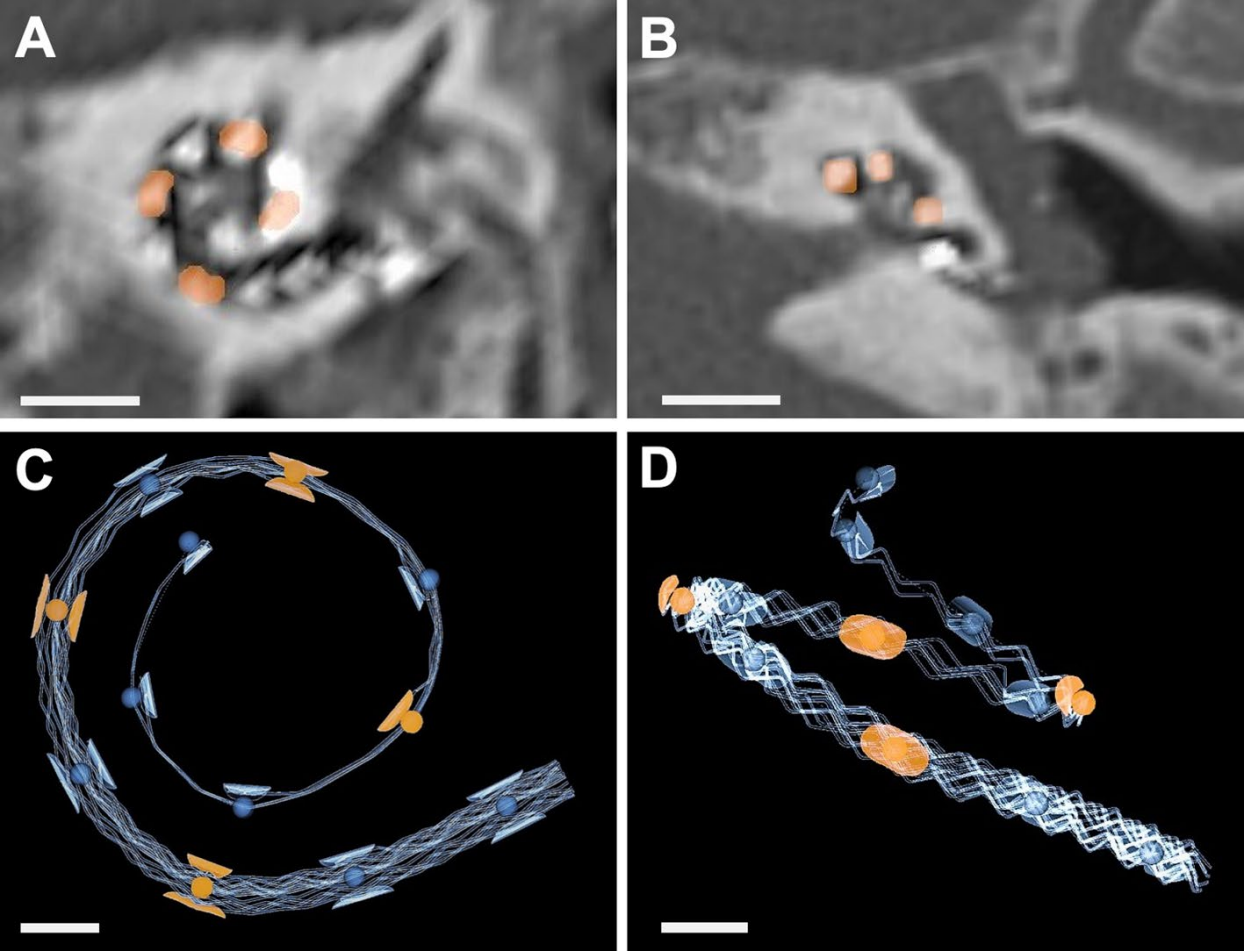
Postoperative angular insertion depth determination. **A** Postsurgical CT-scan oblique coronal view. Electrode array positioned along the lateral cochlear wall. Electrode contacts entering the cochlea 90°, 180°, 270° and 360° are marked in orange. **B** Postsurgical CT-scan axial view. Scale bar 5 mm. **C** Three-dimensional electrode array reconstruction from oblique coronal view. Electrode contacts entering the cochlea 90°, 180°, 270° and 360° are marked in orange. **D** Three-dimensional electrode array reconstruction from axial view. Scale bar 1 mm

The postoperative insertion angle was measured on postoperative high resolution computed tomography (HRCT) imaging control. The round window was localized, and the individual electrode contacts were marked manually (Fig. 1A). The software output is the insertion angle in degree. In addition, the individual electrode contact positioned at 90°, 180°, 270°, 360°, 450° and 540° were determined manually (Fig. 1B).

### ECAP measurements

ECAP measurements were performed using the Med-el fitting software (Maestro version 6.0.1, 7.0.1 and 8.0.1, Med-el GmbH, Innsbruck, Austria). The ECAP stimulation of every individual electrode was recorded. The electrode located apical to the stimulating electrode was used as the recording electrode. When electrode 1 (most apical) was stimulated, electrode 2 (more basal electrode) was used as the recording electrode. The stimulation protocol was chosen as follows: inter phase gap 2.10E−06 s; maximum charge: 35 qu, phase duration 4.00E−05 s. The stimulation charge was increased up to 50 qu when no threshold was detected. For reason of homogeneity, we only used stimulation charges of 35 qu for the sub-analysis of the different insertion angles in degrees.

### Statistical analysis

ECAP amplitude was defined as the voltage difference between the negative N1 peak and the following positive P2 peak. Slope of the ECAP amplitude growth function was calculated at the 50% level. Statistical analyses were performed using Prism (version 8, GraphPad Software, La Jolla, CA, USA). The significance level was set to *p* < 0.05. The assumption of normality was tested graphically using quantile–quantile plots. If not otherwise specified, data are presented as mean with standard deviation (SD) or absolute numbers with percentages. Correlations were assessed using Spearman’s correlation coefficient. For comparison of > 2 groups, a one-way ANOVA was performed.

## Results

**Fig. 2:**
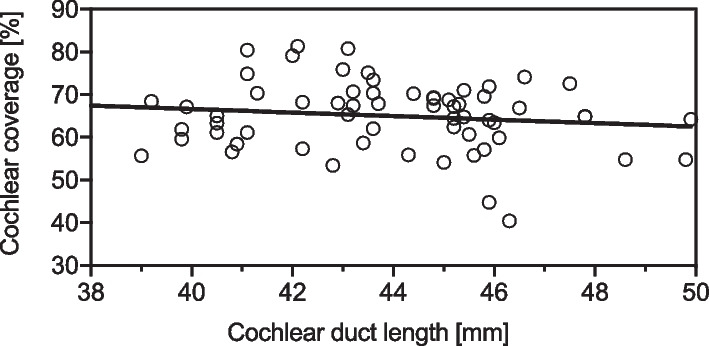
Correlation of cochlear coverage (%) and cochlear duct length. Solid line represents linear regression line. *r*, Spearman’s rank correlation coefficient

A total of 64 ears from 64 CI users (mean age: 64.5 years, SD 7.8 years) were included in the present study. The patients’ demographics are shown in Table 1. The electrode was inserted by round window approach in every case. The round window niche was accessed by mastoidectomy and posterior tympanotomy. Implantation and full insertion of the flexible lateral wall electrode array was achieved in all cases as verified by intraoperative impedance and ECAP measurements as well as by postoperative CT imaging. The mean cochlear duct length was 43.7 mm (SD: 2.8 mm). Mean cochlear coverage (CC) of the electrode array was 65.1% (SD 8.2%) of the CDL (Fig. 2), which corresponds to an average insertion angle of 586 degrees (SD: 73). In 36 out of 64 subjects ECAPs could be measured with a maximum stimulation charge of 35qu, in 45 patients ECAPs were measured with a maximum charge of 50qu.

**Table 1:** Patients’ demographics

	Patients (*n* = 64)
Mean age—years (SD)	64.5 (SD 7.8)
Sex, female:male—*n* (%)	28 (43.8); 36 (56.2)
Side, right:left—*n* (%)	31 ((48.4); 33 (51.6)
Cochlear duct length (mm)	43.7 (2.8)
Cochlear coverage (%)	65.1 (2.8)
Insertion angle (°)	585.7 (73.4)
*Electrode length—n (%)*	
31.5 mm	38 (59.4)
28.0 mm	22 (34.4)
26.0 mm	4 (6.2)

### ECAP measurements

**Fig. 3:**
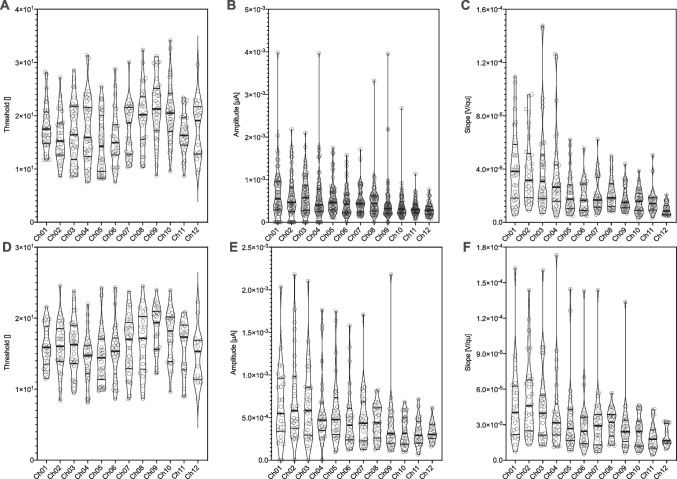
ECAP characteristics and site of stimulation. **A–C** Violin plot of ECAP measured with a maximum stimulation rate of 50 qu. **A** Violin plot of ECAP threshold **B** Violin plot of ECAP amplitude and **C** Violin plot of ECAP slope for each individual stimulated electrode. **D–F** Violin plot of ECAP measured with a maximum stimulation rate of 35 qu. **D** Violin plot of ECAP threshold **E** Violin plot of ECAP amplitude and **F** Violin plot of ECAP slope for each individual stimulated electrode. The “violin” covers the entire range of the datasets with the width indicating frequency, in analogy to a vertical diagram. Bold horizontal line indicates median, soft horizontal lines indicate interquartile ranges

There was a significant effect of electrode location on ECAP threshold, with lower thresholds at the more apical electrodes (*F* (11, 367) = 4,675, *p* < 0.0001; Fig. 3A). ECAP amplitudes were larger towards the apical end of the electrode array (*F* (11, 524) = 2.083, *p* = 0.02; Fig. 3B). The slope of the ECAP amplitude growth function, measured at the 50% level of the growth function also increased towards the apical end of the electrode array *F* (11, 362) = 9.729, *p* < 0.0001; Fig. 3C). The effect was identical when only the subjects were included in whom ECAPs were measured up to a maximum charge of 35qu (Fig. 3D–F).

**Fig. 4:**
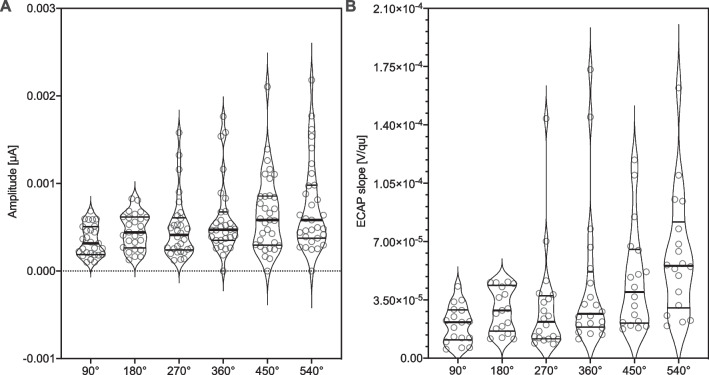
ECAP characteristics and site of stimulation determined from angular electrode positioning. **A** Violin plot of ECAP amplitude and **B** violin plot of ECAP slope for the individual stimulated electrode at 90°, 180°, 270°, 360°, 450° and 540° insertion depth. The “violin” covers the entire range of the datasets with the width indicating frequency, in analogy to a vertical diagram. Bold horizontal line indicates median, soft horizontal lines indicate interquartile ranges

**Fig. 5:**
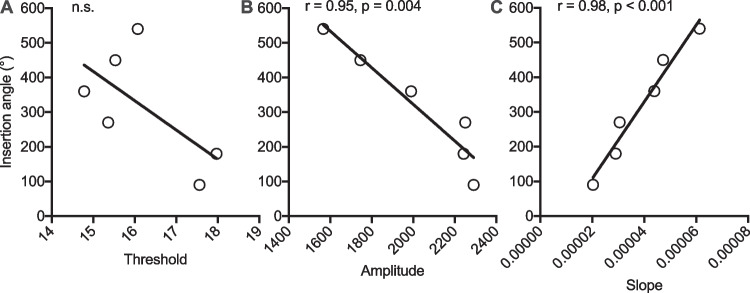
Simple linear regression between the insertion angles at 90°, 180°, 270°, 360°, 450° and 540° and **A** ECAP thresholds, **B** ECAP amplitudes and **C** ECAP slopes. Solid line represents linear regression line. *r*, Spearman’s rank correlation coefficient

Postoperative CT reconstructions of the electrode arrays, revealed an insertion angle of 90° to correspond with electrode 9–12 (range: 3, mean: 10.20, SD: 0.78), 180° with electrode 6–10 (range 4, mean: 7.89, SD: 1.01), 270° with electrode 3–8 (range 5, mean: 5.70, SD: 1.03), 360° with electrode 1–6 (range 5, mean: 4.00, SD: 1.04), 450° with electrode 1–5 (range 4, mean: 3.05, SD: 0.94), 540° with electrode 1–4 (range 3, mean: 2.02, SD: 0.91). Figure 4 shows the increase in ECAP amplitude (*F* (5, 171) = 3.164, *p* = 0.009; Fig. 4A) and slope (*F* (5, 104) = 4,188, *p* < 0.0001; Fig. 4B) with increasing insertion angle. Simple linear regression between the insertion angles at 90°, 180°, 270°, 360°, 450° and 540° showed a significant linearity between these insertion angles and the ECAP slopes (*r* = 0.98, *p* < 0.001) as well as the ECAP amplitudes (*r* = 0.95, *p* = 0.004, Fig. 5).

## Discussion

In the present study, we found a correlation between ECAP characteristics, i.e. threshold, amplitude and slope, and the site of stimulation inside the human cochlea. This is in line with a number of studies reporting variances in ECAP characteristics throughout the cochlea [9, 17–19]. A recent study reported significantly higher ECAP amplitudes throughout the apical and middle regions of the cochlea and significantly lower ECAP thresholds in patients with preserved low-frequency acoustic hearing [2]. Other explanations influencing ECAP characteristics are (1) the decreasing diameter of the cochlear turns towards the apex reducing the distance between the electrode and the neural tissue [20] and (2) an increasing density of neurons in the apical regions [21]. Nevertheless, ECAP may be a promising approach to monitor the electrode’s position during insertion.

Furthermore, the choice of the electrode array length according to the CDL seems to be feasible since there was no correlation between the CDL and the CC. Consequently, when choosing the electrode array according to the CDL homogenous ECAP characteristics among different individuals are observed. However, the mean CC was underestimated by preoperative estimation which is in line with recent studies investigating the accuracy of CC prediction [16, 22].

The findings of this study contribute to optimize existing methodologies to more accurately determine the postoperative insertion angle of the electrode array, without the need of postoperative CT imaging. This has become even more important since approaches to fit the patients speech processor to the CT-based anatomical data, is reported to result in improved hearing outcomes, music appreciation and improvements in quality of life [23–26]. In addition, it might aid in future development of methods for continuous ECAP measurements to monitor and predict the intracochlear electrode array position during surgery. This can be especially relevant for cochlear implantation with the goal of electroacoustic stimulation [9] in which the position of the electrode array is adjusted to the residual hearing.

### Limitations

This study is limited by heterogeneous stimulation strategies. The standard stimulation charge was set to 35 qu. However, in cases of missing thresholds, the charge was increased up to 50 qu. Consequently, comparisons among these different charges are limited. For reason of homogeneity, we only used stimulation charges of 35 qu for the sub-analysis of the different insertion angles in degrees. However, this reduced the number of values that could be analyzed. Thus, the effect of linearity needs to be reproduced in larger cohorts.

## Conclusion

ECAP characteristics are reliably associated with the electrode’s position inside the cochlea. We encourage prospective studies with standardized stimulation protocols to strengthen the results from our study under the aim of identifying standard ECAP values for the site of stimulation.

## Data Availability

Data are available on special request when contacting the corresponding author.
